# Crystal structure and Hirshfeld surface analysis of (3a*RS*,4*RS*,10*SR*,10a*SR*)-2-(3,5-di­methyl­phen­yl)-4-hy­droxy-10-methyl-1-oxo-2,3,3a,4,10,10a-hexa­hydro-1*H*-[1]benzofuro[2,3-*f*]iso­indole-10-carb­oxy­lic acid di­methyl­formamide monosolvate

**DOI:** 10.1107/S2056989025006814

**Published:** 2025-08-07

**Authors:** Elizaveta D. Yakovleva, Victoria I. Salakhova, Victor N. Khrustalev, Roya Z. Nazarova, Khudayar I. Hasanov, Tahir A. Javadzade, Mehmet Akkurt, Gizachew Mulugeta Manahelohe

**Affiliations:** a RUDN University, 6 Miklukho-Maklaya St., Moscow 117198, Russian Federation; b Zelinsky Institute of Organic Chemistry of RAS, 4, 7 Leninsky Prospect, 119991 Moscow, Russian Federation; c Baku Engineering University, Khirdalan, Hasan Aliyev Str. 120, AZ0101, Absheron, Azerbaijan; d Azerbaijan Medical University, Scientific Research Centre (SRC), A. Kasumzade St. 14, AZ 1022, Baku, Azerbaijan; e Department of Chemistry and Chemical Engineering, Khazar University, Baku, Mahsati St. 41, AZ1096, Baku, Azerbaijan; f Department of Physics, Faculty of Sciences, Erciyes University, 38039 Kayseri, Türkiye; g Department of Chemistry, University of Gondar, PO Box 196, Gondar, Ethiopia; Universidade de Sâo Paulo, Brazil

**Keywords:** crystal structure, hydrogen bonds, envelope conformation, Hirshfeld surface analysis

## Abstract

The mol­ecular conformation of the title compound, C_24_H_23_NO_5_·C_3_H_7_NO, is consolidated by intra­molecular C—H⋯O O—H⋯O hydrogen bonds, forming an *S*(6) ring motif. In the crystal, the mol­ecules are connected by C—H⋯O hydrogen bonds, forming layers parallel to the (101) plane. Furthermore, the mol­ecules form layers parallel to the (102) plane by C—H⋯π inter­actions. Important inter­molecular inter­actions highlighted by Hirshfeld surface analysis are H⋯H (54.7%), O⋯H/H⋯O (23.0%), and C⋯H/H⋯C (19.9%) contacts.

## Abstract 2

The mol­ecular conformation of the title compound, C_24_H_23_NO_5_·C_3_H_7_NO, is consolidated by intra­molecular C—H⋯O O—H⋯O hydrogen bonds, forming an *S*(6) ring motif. In the crystal, the mol­ecules are connected by C—H⋯O hydrogen bonds, forming layers parallel to the (101) plane. Additionally, C—H⋯π inter­actions lead to the formation of layers parallel to the (102) plane.

## 1. Chemical context

**Figure 1:**
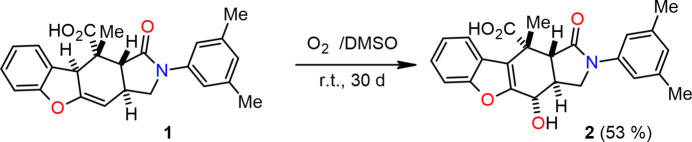
Synthesis of (3a*RS*,4*RS*,10*SR*,10*aSR*)-2-(3,5-di­methyl­phen­yl)-4-hy­droxy-10-methyl-1-oxo-2,3,3a,4,10,10*a*-hexa­hydro-1*H*-[1]benzofuro[2,3-*f*]iso­indole-10-carb­oxy­lic acid (**2**).

The IMDAV reaction (Intra-Mol­ecular Diels–Alder in Vinyl­heteroarenes) is a useful tool for the one-step synthesis of benzo­furans, indoles, benzo­thio­phenes, and pyrrolo­pyridines annulated with other carbocycles and heterocycles (Horak *et al.*, 2017 ▸; Krishna *et al.*, 2022 ▸; Nadirova *et al.*, 2020 ▸; Shelukho *et al.*, 2025 ▸; Yakovleva *et al.*, 2024 ▸; Zaytsev *et al.*, 2023 ▸, 2025 ▸; Zubkov *et al.*, 2016 ▸). In a continuation of our research on the properties of vinyl­heteroarene systems previously obtained *via* tandem acyl­ation/[4 + 2] cyclo­addition between 3-(heteroar­yl)allyl­amines and maleic anhydrides, an example of an IMDAV reaction, we present here the second instance of spontaneous slow oxidation of adduct **1** (Fig. 1 ▸) in DMSO under aerobic conditions. Previous studies have shown that benzothienoisoindolones of type **1** undergo oxidation when stored for a long time in DMSO at room temperature (Mammadova *et al.*, 2023 ▸). Presumably, the DMSO acts as a mild oxidant, as observed in several other oxidation reactions, including the Pfitzner–Moffatt, Corey–Kim, Swern, and Kornblum oxidations (Epstein *et al.*, 1967 ▸).

Slow oxidation of (3a*RS*,9b*RS*,10*RS*,10a*SR*)-2-(3,5-di­methyl­phen­yl)-10-methyl-1-oxo-2,3,3a,9b,10,10a-hexa­hydro-1*H*-[1]benzofuro[2,3-*f*]iso­indole-10-carb­oxy­lic acid (**1**) occurs when the solution is stirred in dimethyl sulfoxide (DMSO) for one month at r.t. The title compound **2** was isolated in a 53% yield after standard treatment of the reaction mixture followed by recrystallization from an EtOH/DMF mixture. As in the previous case (Mammadova *et al.*, 2023 ▸), the reaction does not stop at the formation of an alcohol. This leads to the formation of the aromatic product **2** as a result of proton migration.
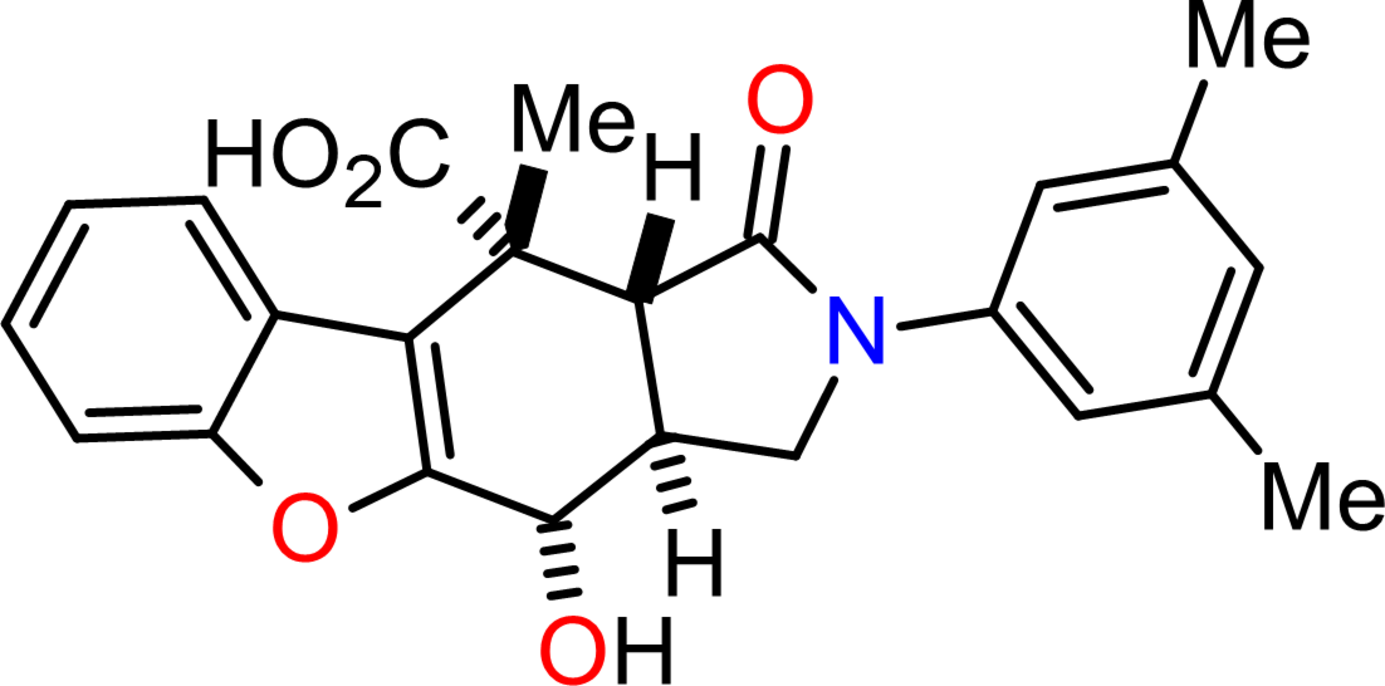


## 2. Structural commentary

**Figure 2:**
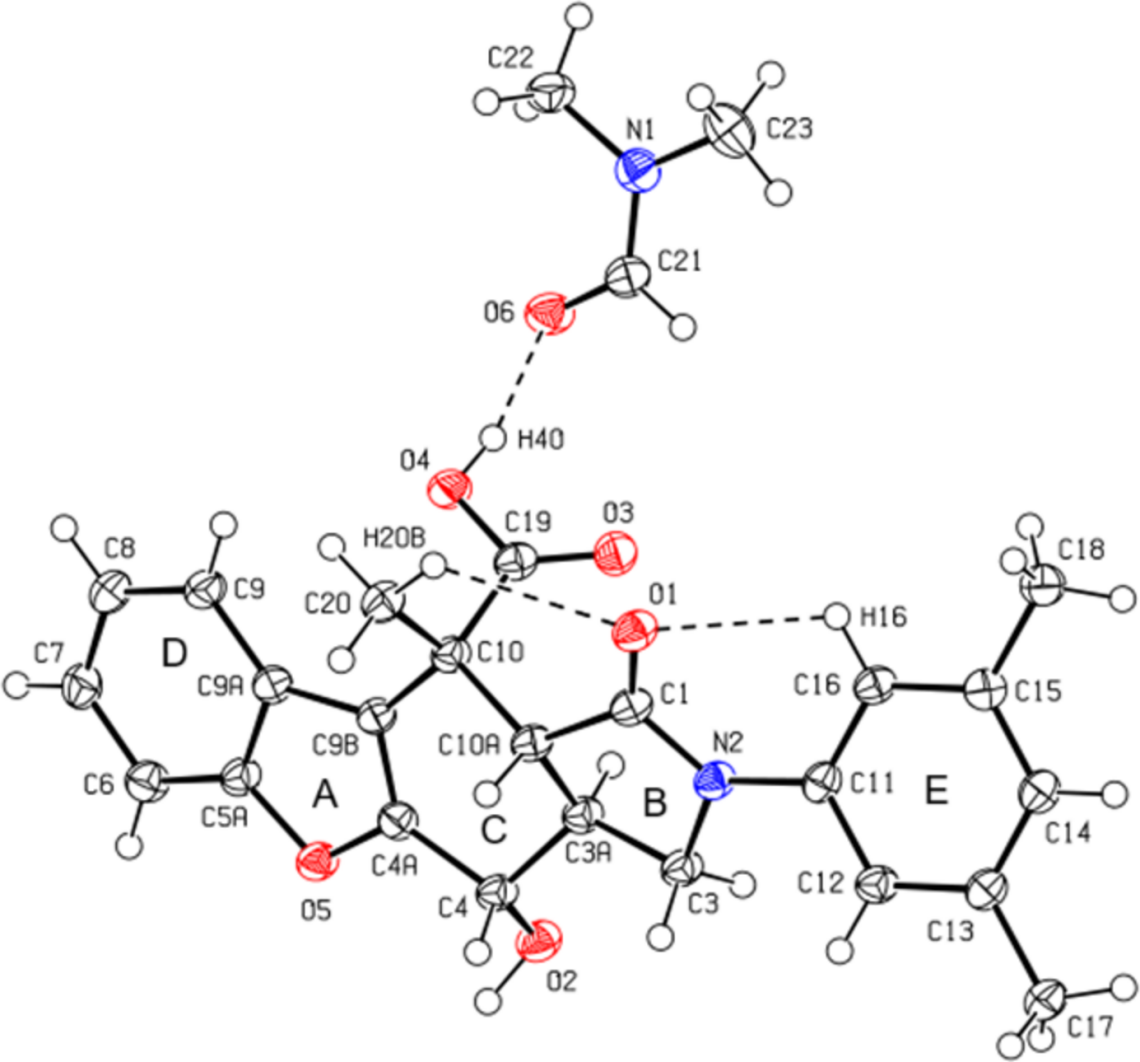
View of the title mol­ecule. Displacement ellipsoids are drawn at the 50% probability level.

The mol­ecular conformation of the title compound is consolidated by intra­molecular C—H⋯O hydrogen bonds and intra­molecular O—H⋯O hydrogen bonds, forming an *S*(6) ring motif (Fig. 2 ▸; Table 1 ▸; Bernstein *et al.*, 1995 ▸). The main mol­ecule of the title compound is planar, with a mean deviation of 0.002 Å from the least-squares plane defined by the 53 atoms (excluding H atoms). The deviations of some atoms from the least-squares plane are 1.141 (2) Å for O1, −1.083 (2) Å for O2, −1.414 (2) Å for O3, −1.224 (2) Å for O4, 0.734 (2) Å for C1, 0.718 (2) Å for C10*A*, −0.631 (2) Å for C18, −0.841 (2) Å for C19 and 1.585 (2) Å for C20. The five-membered *B* (N2/C1/C10A/C3A/C3) ring adopts an envelope conformation, as indicated by the puckering parameters (Cremer & Pople, 1975 ▸) *Q*(2) = 0.337 (2) Å, φ(2) = 287.7 (4)°, with the C3*A* atom −0.212 (2) Å out of the plane defined by the other atoms of the main mol­ecule. The six-membered *C* (C3*A*/C4/C4*A*/C9*B*/C10/C10*A*) ring has a half-chair conformation [the puckering parameters are *Q*_T_ = 0.533 (2) Å, θ = 127.0 (2)°, and φ = 152.1 (3)°]. The dihedral angles between the least-squares planes of the rings in the mol­ecule are *A*/*B* = 19.28 (12), *A*/*C* = 6.72 (11), *A*/*D* = 2.10 (11), *A*/*E* = 19.19 (11), *B*/*C* = 12.57 (11), *B*/*D* = 20.45 (12), *B*/*E* = 35.55 (11), *C*/*D* = 8.10 (10), *C*/*E* = 24.24 (10) and *D*/*E* = 19.75 (10)°. There is one stereogenic center in the title mol­ecule and the chirality about atom C23 is *S* in the chosen asymmetric unit. The geometric properties of the title compound are normal and consistent with those of related compounds listed in the *Database survey* section.

## 3. Supra­molecular features and Hirshfeld surface analysis

**Figure 3:**
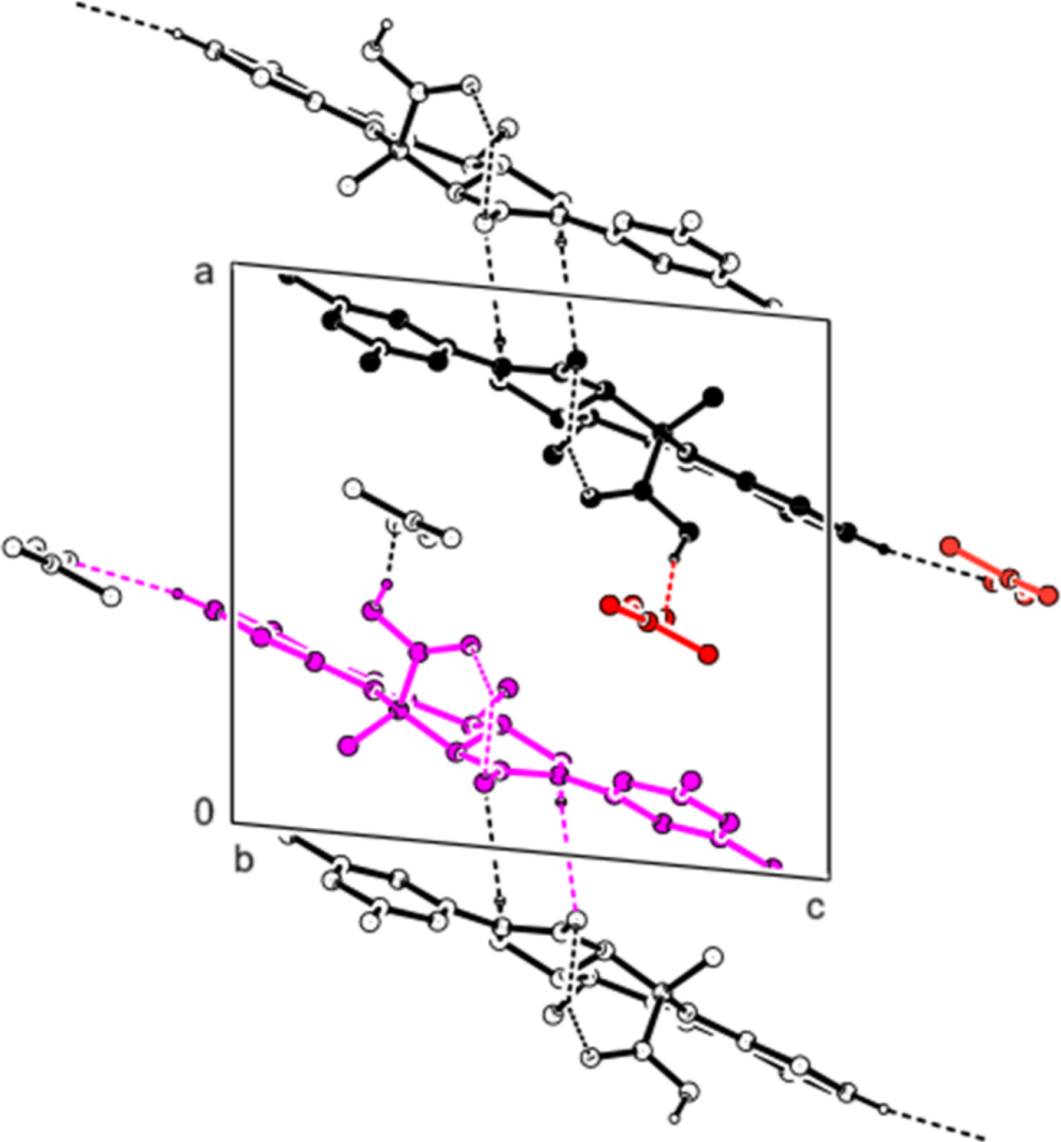
A partial view of the mol­ecular packing along the *b* axis, showing the O—H⋯O and C—H⋯O inter­actions.

**Figure 4:**
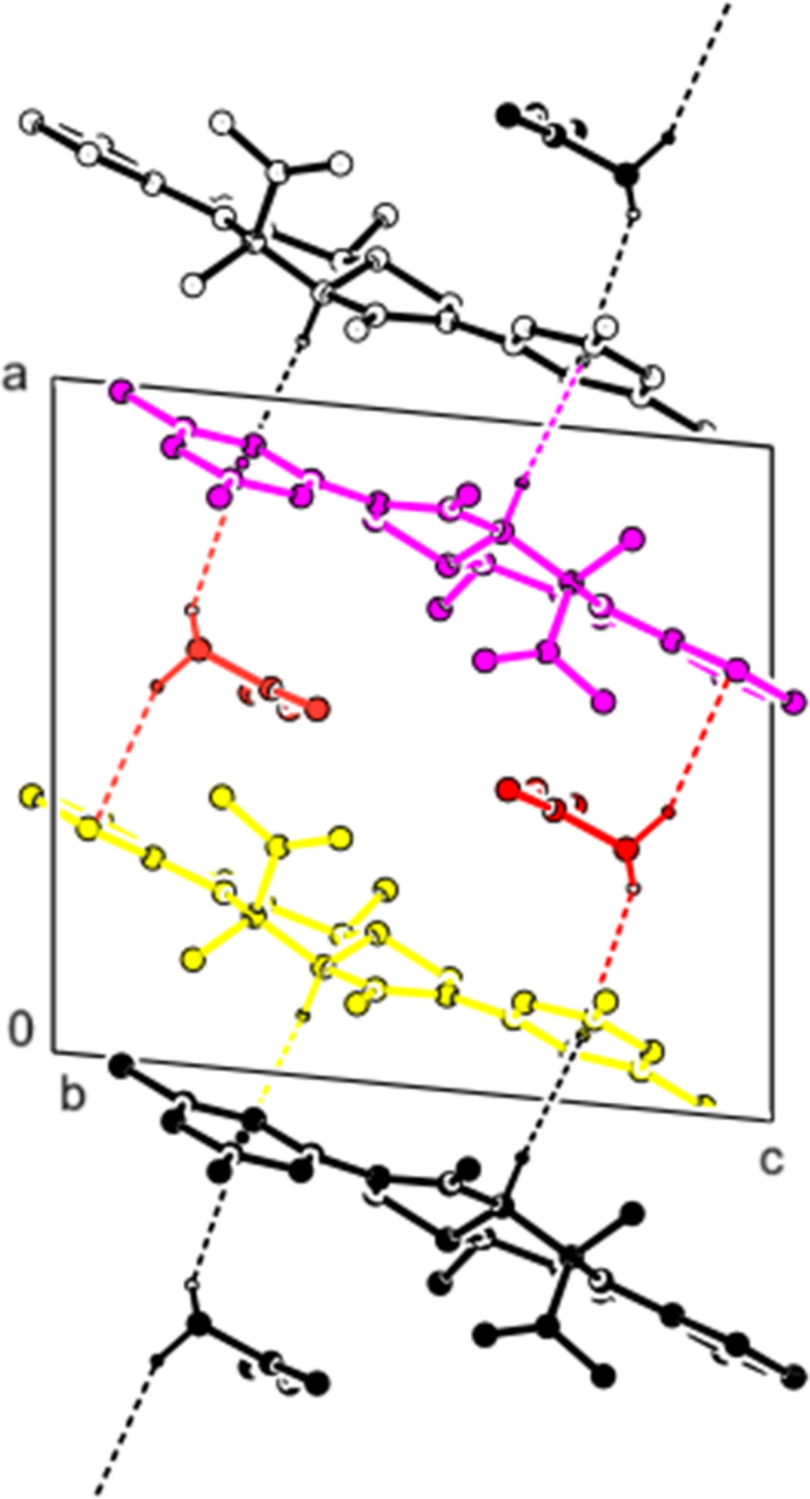
A partial view of the mol­ecular packing along the *b* axis, showing the C—H⋯π inter­actions.

In the crystal, the mol­ecules are connected by C—H⋯O hydrogen bonds, forming layers parallel to the (101) plane (Table 1 ▸; Fig. 3 ▸). Furthermore, the mol­ecules form layers parallel to the (10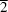) plane by C—H⋯π inter­actions (Table 1 ▸; Fig. 4 ▸. No π–π inter­actions were observed.

**Figure 5:**
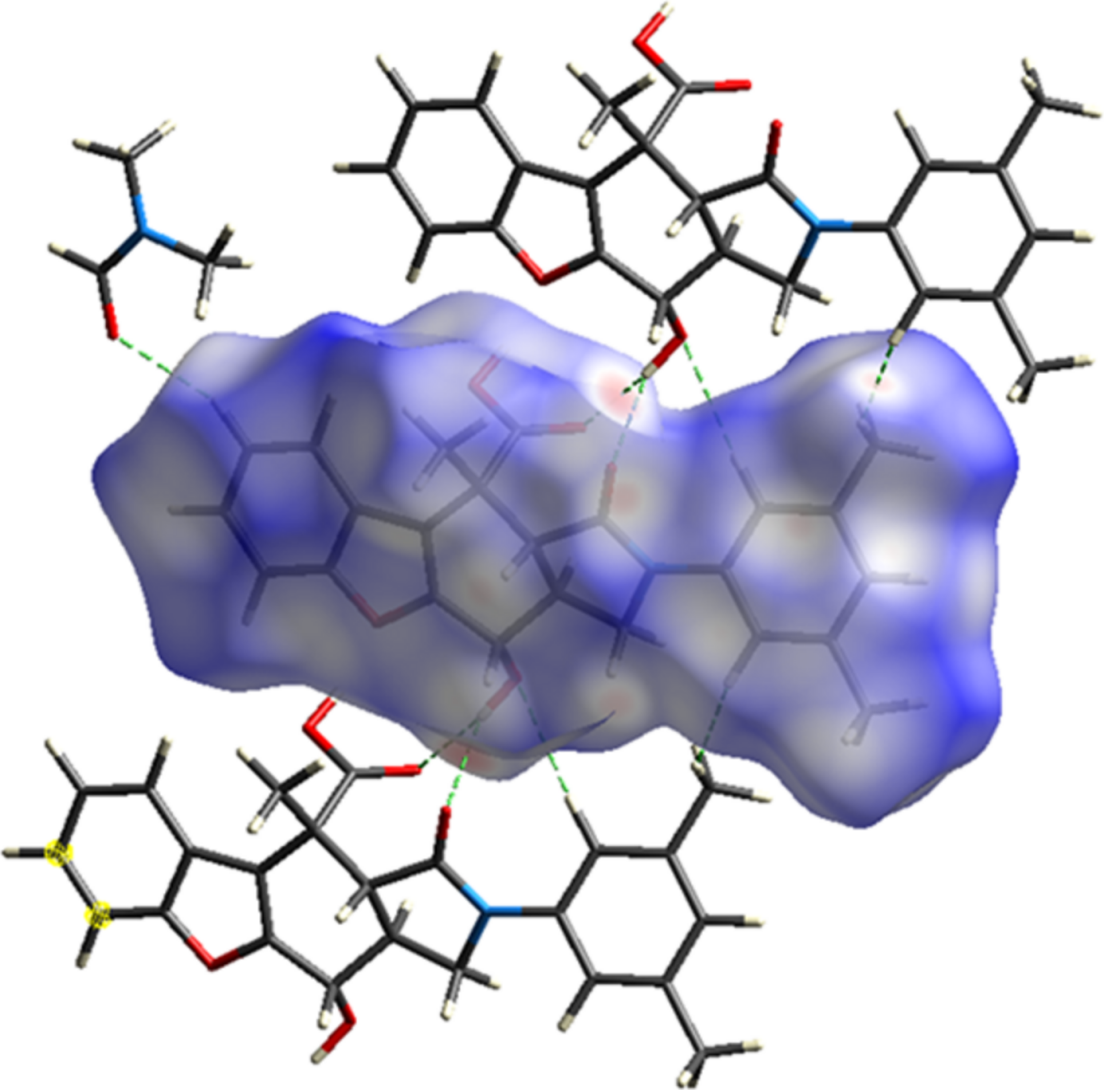
Hirshfeld surface of the title compound mapped with *d*_norm_.

*CrystalExplorer 17.5* (Spackman *et al.*, 2021 ▸) was used to construct Hirshfeld surfaces and generate the related two-dimensional fingerprint plots to illustrate the inter­molecular inter­actions for the mol­ecules of the title compound. The *d*_norm_ mappings of the title compound were conducted in the range −0.7845 to +1.3229 a.u. Bright-red circles on the *d*_norm_ surfaces (Fig. 5 ▸) represent H⋯H, O—H⋯O and C—H⋯O inter­action zones (Tables 1 ▸ and 2 ▸).

**Figure 6:**
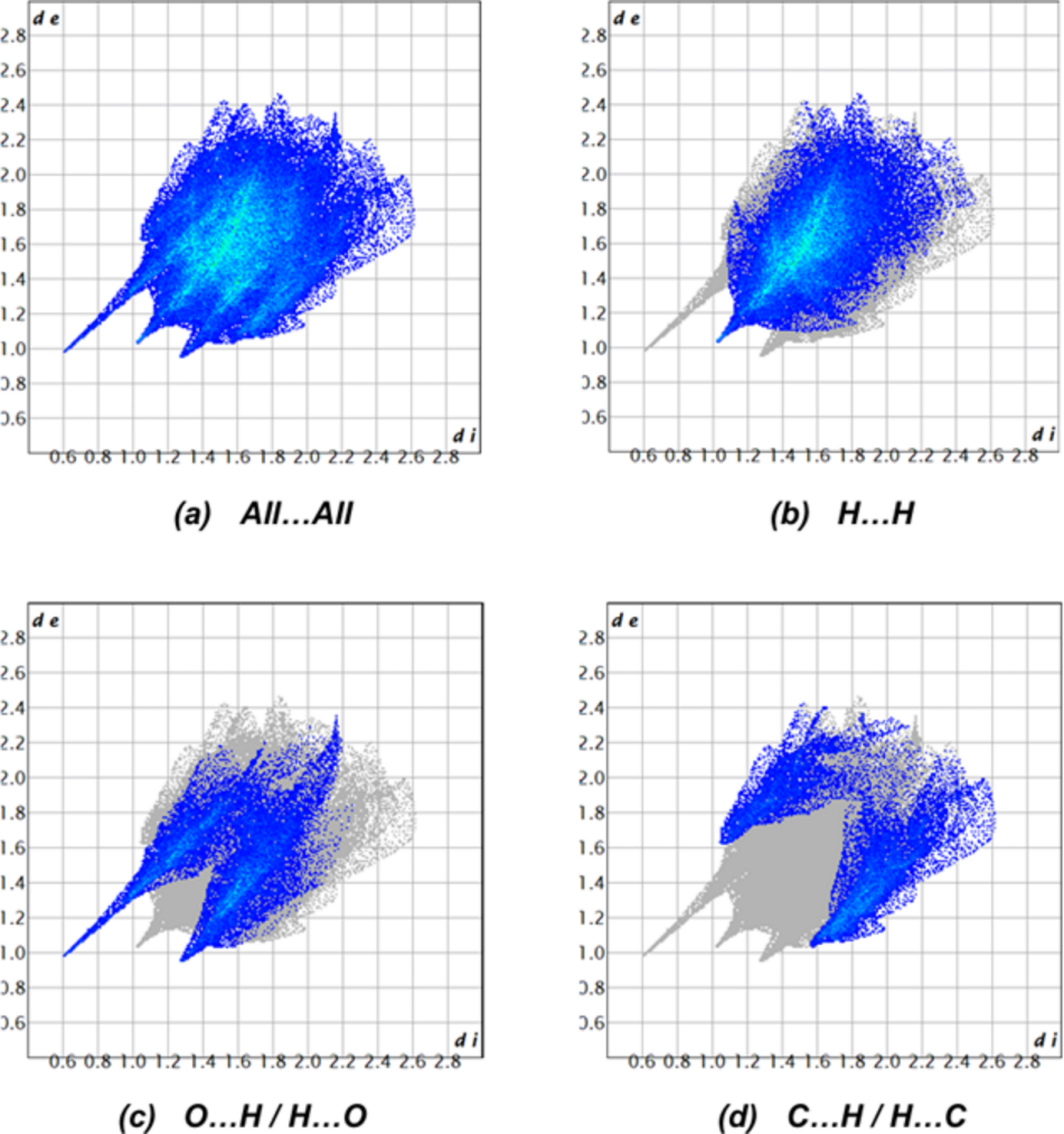
The two-dimensional fingerprint plots for the compound showing (*a*) all inter­actions, and delineated into (*b*) H⋯H (54.7%), (*c*) O⋯H/H⋯O (23.0%) and (*d*) C⋯H/H⋯C (19.9%) inter­actions. The *d*_i_ and *d*_e_ values are the closest inter­nal and external distances (in Å) from given points on the Hirshfeld surface.

Two-dimensional fingerprint plots together with their percentage contributions are shown in Fig. 6 ▸. The crystal packing is dominated by H⋯H contacts, representing van der Waals inter­actions (54.7% contribution to the overall surface), followed by O⋯H/H⋯O and C⋯H/H⋯C inter­actions, which contribute to 23.0% and 19.9%, respectively. The other contacts (N⋯H/H⋯N 0.7%, O⋯C/C⋯O 0.6%, C⋯C 0.4%, O⋯O 0.3%, N⋯C/C⋯N 0.2%, O⋯N/N⋯O 0.1% and N⋯N 0.1%) only make a minor contribution to the crystal packing.

## 4. Database survey

A search of the Cambridge Structural Database (CSD, version 6.00, update April 2025; Groom *et al.*, 2016 ▸) for the *octa­hydro-1H-isoindol-1-one* unit gave 467 hits. The five related compound CSD reference codes are ANAMUZ (Mariaule *et al.*, 2016 ▸), BAFYAL (Zhong *et al.*, 2017 ▸), NAMROK (Chou & Wu, 2012 ▸), TODKEF (Elliott & Booker-Milburn, 2019 ▸) and YOPXIL (Paddon-Row *et al.*, 2009 ▸).

ANAMUZ crystallizes in the monoclinic *P*2_1_/*c* space group, BAFYAL in the ortho­rhom­bic *Pna*2_1_ space group, NAMROK in the monoclinic *P*2_1_/*n* space group, TODKEF in the monoclinic *C*2/*c* space group, and YOPXIL in the monoclinic *P*2_1_ like the title compound.

In the structure of ANAMUZ, the mol­ecules are linked by C—H⋯O and O—H⋯O inter­molecular hydrogen bonds, forming a three-dimensional network. Weak π–π inter­actions are also observed. In BAFYAL, the mol­ecules are linked by C—H⋯O inter­actions, forming layers parallel to the (002) plane. π–π inter­actions are also present. In NAMROK, pairs of mol­ecules are linked by C—H⋯O inter­actions. π–π and C—H⋯π inter­actions are not observed. In TODKEF, the mol­ecules are linked by inter­molecular C—H⋯O and O—H⋯O hydrogen bonds, forming a three-dimensional network. C—H⋯π inter­actions are also observed. In YOPXIL, the mol­ecules are linked by inter­molecular C—H⋯O hydrogen bonds, forming chains along the *b*-axis direction. No π–π or C—H⋯π inter­actions are observed.

## 5. Synthesis and crystallization

A solution of (3a*RS*,9b*RS*,10*RS*,10a*SR*)-2-(3,5-di­methyl­phen­yl)-10-methyl-1-oxo-2,3,3a,9b,10,10a-hexa­hydro-1*H*-[1]benzofuro[2,3-*f*]iso­indole-10-carb­oxy­lic acid **1** (39.0 mg, 0.1 mmol) in 0.5 mL of DMSO was stirred for 30 d in an open flask. The reaction mixture was concentrated, recrystallized from a mixture of EtOH/DMF. The solid was filtered off, washed with Et_2_O (3 × 1 mL), and air dried. The title compound was obtained as a colorless plates, yield 53%, 21.5 mg; m.p. > 523 K (with decomp.). IR (KBr), *ν* (cm^−1^): 3047 (OH), 1744 (CO_2_), 1683 (N—C=O). ^1^H NMR (700.2 MHz, DMSO-*d_6_*): *δ* (*J*, Hz) 12.87 (*s*, 1H, CO_2_H), 7.30–7.22 (*m*, 4H, H Ar), 7.09–7.06 (*m*, 2H, H Ar), 6.76 (*br.s*, 1H, H Ar), 5.69 (*br.s*, 1H, OH), 4.31 (*br.s*, 1H, H-4) 4.02 (*t*, *J* = 8.6, 1H, H-3A), 3.69 (*t*, *J* = 8.6, 1H, H-3B), 3.03–2.98 (*m*, 1H, H-3a), 2.26 (*s*, 6H, CH_3_), 2.14 (*d*, *J* = 12.6, 1H, H-10a), 0.99 (*s*, 3H, CH_3_) ppm. ^13^C{^1^H} NMR (176.1 MHz, DMSO-*d_6_*): *δ* 177.3, 172.6, 158.8, 156.9, 140.2, 138.2 (2C), 129.4, 126.4, 125.8, 125.4, 122.8, 117.6 (2C), 110.6, 98.8, 58.9, 50.3, 49.7, 42.9, 35.9, 22.8, 21.7 (2C) ppm. MS (ESI) *m*/*z*: [*M* + H]^+^ 406. Elemental analysis calculated (%) for C_24_H_23_NO_5_·C_3_H_7_NO: C 67.77, H 6.32, N 5.85; found: C 68.04, H 6.49, N 6.01.

## 6. Refinement

Crystal data, data collection and structure refinement details are summarized in Table 3 ▸. The hydroxyl H atoms were found in the difference Fourier maps [O2—H2*O* = 0.99 (5) and O4—H4*O* = 0.84 (4) Å] and refined with *U*_iso_(H) = 1.5*U*_eq_(O). All C-bound H atoms were positioned geometrically (C—H = 0.95 and 1.00 Å) and refined using a riding model with *U*_iso_(H) = 1.2 or 1.5*U*_eq_(C). Owing to poor agreement between observed and calculated intensities, two outliers (\-13 \-5 3 and 14 3 2) were omitted in the final cycles of refinement.

## Supplementary Material

Crystal structure: contains datablock(s) I. DOI: 10.1107/S2056989025006814/ex2094sup1.cif

Structure factors: contains datablock(s) I. DOI: 10.1107/S2056989025006814/ex2094Isup2.hkl

Supporting information file. DOI: 10.1107/S2056989025006814/ex2094Isup3.cml

CCDC reference: 2477243

Additional supporting information:  crystallographic information; 3D view; checkCIF report

## Figures and Tables

**Table 1:** Hydrogen-bond geometry (Å, °) *Cg*4 and *Cg*5 are the centroids of the C5*A*/C6–C9/C9*A* and C11–C16 rings, respectively.

*D*—H⋯*A*	*D*—H	H⋯*A*	*D*⋯*A*	*D*—H⋯*A*
O2—H2*O*⋯O1^i^	0.99 (5)	2.23 (4)	3.013 (2)	135 (3)
O2—H2*O*⋯O3^i^	0.99 (5)	2.32 (4)	3.152 (3)	141 (3)
O4—H4*O*⋯O6	0.84 (4)	1.74 (4)	2.560 (3)	165 (4)
C3—H3*A*⋯O1^ii^	0.99	2.54	3.452 (3)	152
C8—H8⋯O6^iii^	0.95	2.44	3.344 (3)	159
C16—H16⋯O1	0.95	2.44	2.970 (3)	115
C20—H20*B*⋯O1	0.98	2.59	3.229 (3)	123
C10*A*—H10*A*⋯*Cg*5^ii^	1.00	2.88	3.865 (2)	169
C22—H22*A*⋯*Cg*5^iv^	0.98	2.96	3.401 (3)	109
C22—H22*C*⋯*Cg*4^v^	0.98	2.68	3.594 (3)	155

Symmetry codes: (i) 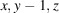; (ii) 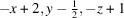; (iii) 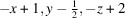; (iv) 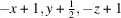; (v) 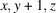.

**Table 2:** Summary of short inter­atomic contacts (Å)

Contact	Distance	Symmetry operation
O1⋯H2*O*	2.23	*x*, 1 + *y*, *z*
O1⋯H3*A*	2.54	2 − *x*, 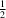 + *y*, 1 − *z*
O4⋯H23*A*	2.60	*x*, −1 + *y*, *z*
H2*O*⋯H23*B*	2.55	1 − *x*, − 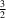 + *y*, 1 − *z*
H4*O*⋯O6	1.74	*x*, *y*, *z*
H16⋯H23*B*	2.58	1 − *x*, − 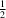 + *y*, 1 − *z*
C7⋯H18*B*	3.04	*x*, −1 + *y*, 1 + *z*
H8⋯O6	2.44	1 − *x*, − 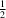 + *y*, 2 − *z*
H7⋯H22*B*	2.52	1 − *x*, − 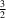 + *y*, 2 − *z*

**Table 3:** Experimental details

Crystal data	
Chemical formula	C_24_H_23_NO_5_·C_3_H_7_NO
*M*_r_	478.53
Crystal system, space group	Monoclinic, *P*2_1_
Temperature (K)	100
*a*, *b*, *c* (Å)	11.88334 (13), 7.80196 (10), 12.71675 (15)
β (°)	95.5166 (10)
*V* (Å^3^)	1173.55 (2)
*Z*	2
Radiation type	Cu *K*α
μ (mm^−1^)	0.79
Crystal size (mm)	0.32 × 0.18 × 0.04

Data collection	
Diffractometer	Rigaku XtaLAB Synergy-S, HyPix-6000HE area-detector
Absorption correction	Multi-scan (*CrysAlis PRO*; Rigaku OD, 2021 ▸)
*T*_min_, *T*_max_	0.840, 1.000
No. of measured, independent and observed [*I* > 2σ(*I*)] reflections	16579, 4632, 4504
*R*_int_	0.039
(sin θ/λ)_max_ (Å^−1^)	0.639

Refinement	
*R*[*F*^2^ > 2σ(*F*^2^)], *wR*(*F*^2^), *S*	0.039, 0.104, 1.05
No. of reflections	4632
No. of parameters	327
No. of restraints	1
H-atom treatment	H atoms treated by a mixture of independent and constrained refinement
Δρ_max_, Δρ_min_ (e Å^−3^)	0.29, −0.20
Absolute structure	Flack *x* determined using 1816 quotients [(*I*^+^)−(*I*^−^)]/[(*I*^+^)+(*I*^−^)] (Parsons *et al.*, 2013 ▸)
Absolute structure parameter	0.26 (7)

Computer programs: *CrysAlis PRO* (Rigaku OD, 2021 ▸), *SHELXT* (Sheldrick, 2015*a* ▸), *SHELXL* (Sheldrick, 2015*b* ▸), *ORTEP-3 for Windows* (Farrugia, 2012 ▸) and *PLATON* (Spek, 2020 ▸).

## supplementary crystallographic information

### (3aRS,4RS,10SR,10aSR)-2-(3,5-Dimethylphenyl)-4-hydroxy-10-methyl-1-oxo-2,3,3a,4,10,10a-hexahydro-1H-[1]benzofuro[2,3-f]isoindole-10-carboxylic acid dimethylformamide monosolvate Crystal data

**Table:**

C_24_H_23_NO_5_·C_3_H_7_NO	*F*(000) = 508
*M**_r_* = 478.53	*D*_x_ = 1.354 Mg m^−^^3^
Monoclinic, *P*2_1_	Cu *K*α radiation, λ = 1.54184 Å
*a* = 11.88334 (13) Å	Cell parameters from 11292 reflections
*b* = 7.80196 (10) Å	θ = 3.5–79.3°
*c* = 12.71675 (15) Å	µ = 0.79 mm^−^^1^
β = 95.5166 (10)°	*T* = 100 K
*V* = 1173.55 (2) Å^3^	Plate, colourless
*Z* = 2	0.32 × 0.18 × 0.04 mm

### (3aRS,4RS,10SR,10aSR)-2-(3,5-Dimethylphenyl)-4-hydroxy-10-methyl-1-oxo-2,3,3a,4,10,10a-hexahydro-1H-[1]benzofuro[2,3-f]isoindole-10-carboxylic acid dimethylformamide monosolvate Data collection

**Table:**

Rigaku XtaLAB Synergy-S, HyPix-6000HE area-detector diffractometer	4504 reflections with *I* > 2σ(*I*)
Radiation source: micro-focus sealed X-ray tube	*R*_int_ = 0.039
φ and ω scans	θ_max_ = 80.0°, θ_min_ = 3.5°
Absorption correction: multi-scan (CrysAlisPro; Rigaku OD, 2021)	*h* = −15→14
*T*_min_ = 0.840, *T*_max_ = 1.000	*k* = −9→9
16579 measured reflections	*l* = −16→16
4632 independent reflections	

### (3aRS,4RS,10SR,10aSR)-2-(3,5-Dimethylphenyl)-4-hydroxy-10-methyl-1-oxo-2,3,3a,4,10,10a-hexahydro-1H-[1]benzofuro[2,3-f]isoindole-10-carboxylic acid dimethylformamide monosolvate Refinement

**Table:**

Refinement on *F*^2^	Hydrogen site location: mixed
Least-squares matrix: full	H atoms treated by a mixture of independent and constrained refinement
*R*[*F*^2^ > 2σ(*F*^2^)] = 0.039	*w* = 1/[σ^2^(*F*_o_^2^) + (0.0664*P*)^2^ + 0.2084*P*] where *P* = (*F*_o_^2^ + 2*F*_c_^2^)/3
*wR*(*F*^2^) = 0.104	(Δ/σ)_max_ < 0.001
*S* = 1.05	Δρ_max_ = 0.29 e Å^−^^3^
4632 reflections	Δρ_min_ = −0.20 e Å^−^^3^
327 parameters	Absolute structure: Flack *x* determined using 1816 quotients [(*I*^+^)-(*I*^-^)]/[(*I*^+^)+(*I*^-^)] (Parsons *et al.*, 2013)
1 restraint	Absolute structure parameter: 0.26 (7)

### (3aRS,4RS,10SR,10aSR)-2-(3,5-Dimethylphenyl)-4-hydroxy-10-methyl-1-oxo-2,3,3a,4,10,10a-hexahydro-1H-[1]benzofuro[2,3-f]isoindole-10-carboxylic acid dimethylformamide monosolvate Special details

**Table:**

Geometry. All esds (except the esd in the dihedral angle between two l.s. planes) are estimated using the full covariance matrix. The cell esds are taken into account individually in the estimation of esds in distances, angles and torsion angles; correlations between esds in cell parameters are only used when they are defined by crystal symmetry. An approximate (isotropic) treatment of cell esds is used for estimating esds involving l.s. planes.

### (3aRS,4RS,10SR,10aSR)-2-(3,5-Dimethylphenyl)-4-hydroxy-10-methyl-1-oxo-2,3,3a,4,10,10a-hexahydro-1H-[1]benzofuro[2,3-f]isoindole-10-carboxylic acid dimethylformamide monosolvate Fractional atomic coordinates and isotropic or equivalent isotropic displacement parameters (Å^2^)

**Table:**

	*x*	*y*	*z*	*U*_iso_*/*U*_eq_	
O1	0.88530 (14)	0.6262 (2)	0.57739 (13)	0.0271 (3)	
O2	0.71225 (14)	−0.0932 (2)	0.53774 (13)	0.0284 (3)	
H2O	0.730 (3)	−0.211 (6)	0.563 (3)	0.043*	
O3	0.64231 (13)	0.5337 (3)	0.60067 (13)	0.0305 (4)	
O4	0.59786 (13)	0.4918 (2)	0.76539 (13)	0.0273 (3)	
H4O	0.548 (3)	0.562 (6)	0.741 (3)	0.041*	
C1	0.86110 (16)	0.4789 (3)	0.55174 (17)	0.0224 (4)	
N2	0.85953 (15)	0.4116 (3)	0.45216 (14)	0.0226 (4)	
C3	0.83569 (18)	0.2262 (3)	0.44817 (17)	0.0238 (4)	
H3A	0.906192	0.158378	0.448925	0.029*	
H3B	0.784816	0.195658	0.384677	0.029*	
C3A	0.77808 (17)	0.1988 (3)	0.54918 (17)	0.0227 (4)	
H3C	0.696408	0.229798	0.534790	0.027*	
C4	0.78611 (17)	0.0212 (3)	0.59804 (17)	0.0224 (4)	
H4	0.865679	−0.021613	0.601253	0.027*	
C4A	0.75155 (16)	0.0443 (3)	0.70768 (16)	0.0222 (4)	
O5	0.72355 (13)	−0.0995 (2)	0.76165 (13)	0.0249 (3)	
C5A	0.69017 (17)	−0.0398 (3)	0.85613 (17)	0.0235 (4)	
C6	0.65086 (19)	−0.1429 (3)	0.93339 (19)	0.0274 (5)	
H6	0.646651	−0.264079	0.926576	0.033*	
C7	0.6179 (2)	−0.0579 (3)	1.02159 (19)	0.0290 (5)	
H7	0.590681	−0.122544	1.077164	0.035*	
C8	0.6241 (2)	0.1209 (4)	1.03023 (19)	0.0293 (5)	
H8	0.600598	0.174756	1.091446	0.035*	
C9	0.66374 (19)	0.2215 (3)	0.95132 (18)	0.0266 (4)	
H9	0.667526	0.342649	0.957983	0.032*	
C9A	0.69807 (17)	0.1389 (3)	0.86142 (17)	0.0227 (4)	
C9B	0.73912 (17)	0.1912 (3)	0.76232 (16)	0.0215 (4)	
C10	0.76988 (17)	0.3675 (3)	0.72104 (17)	0.0217 (4)	
C10A	0.83541 (16)	0.3314 (3)	0.62441 (17)	0.0221 (4)	
H10A	0.909918	0.281612	0.652289	0.027*	
C11	0.88384 (16)	0.5005 (3)	0.35935 (16)	0.0225 (4)	
C12	0.92752 (18)	0.4062 (3)	0.27861 (18)	0.0246 (4)	
H12	0.946899	0.289074	0.289792	0.029*	
C13	0.94276 (17)	0.4833 (3)	0.18188 (17)	0.0247 (4)	
C14	0.91430 (18)	0.6547 (3)	0.16710 (18)	0.0254 (4)	
H14	0.922624	0.706973	0.100812	0.030*	
C15	0.87377 (18)	0.7520 (3)	0.24734 (18)	0.0248 (4)	
C16	0.85946 (17)	0.6739 (3)	0.34459 (17)	0.0231 (4)	
H16	0.833172	0.739347	0.400308	0.028*	
C17	0.9891 (2)	0.3806 (3)	0.09481 (19)	0.0310 (5)	
H17A	1.067808	0.349135	0.116298	0.047*	
H17B	0.985651	0.449642	0.030209	0.047*	
H17C	0.943878	0.276417	0.081523	0.047*	
C18	0.8463 (2)	0.9381 (3)	0.2310 (2)	0.0306 (5)	
H18A	0.772745	0.962733	0.256594	0.046*	
H18B	0.843368	0.965518	0.155612	0.046*	
H18C	0.904707	1.007900	0.270236	0.046*	
C19	0.66366 (17)	0.4730 (3)	0.68735 (17)	0.0235 (4)	
C20	0.84293 (18)	0.4711 (3)	0.80565 (18)	0.0261 (4)	
H20A	0.800078	0.490770	0.866655	0.039*	
H20B	0.863104	0.581564	0.775894	0.039*	
H20C	0.911934	0.406868	0.828098	0.039*	
O6	0.43899 (15)	0.7105 (3)	0.72511 (14)	0.0318 (4)	
N1	0.43032 (15)	0.9966 (3)	0.69699 (16)	0.0285 (4)	
C21	0.45729 (18)	0.8385 (3)	0.67122 (18)	0.0273 (5)	
H21	0.492584	0.821996	0.608054	0.033*	
C22	0.3827 (2)	1.0261 (4)	0.7974 (2)	0.0337 (5)	
H22A	0.325742	0.938282	0.807445	0.051*	
H22B	0.347375	1.139676	0.796540	0.051*	
H22C	0.443070	1.020126	0.855480	0.051*	
C23	0.4538 (2)	1.1426 (4)	0.6328 (2)	0.0374 (6)	
H23A	0.504688	1.221382	0.674296	0.056*	
H23B	0.382962	1.201749	0.609764	0.056*	
H23C	0.489733	1.103704	0.570820	0.056*	

### (3aRS,4RS,10SR,10aSR)-2-(3,5-Dimethylphenyl)-4-hydroxy-10-methyl-1-oxo-2,3,3a,4,10,10a-hexahydro-1H-[1]benzofuro[2,3-f]isoindole-10-carboxylic acid dimethylformamide monosolvate Atomic displacement parameters (Å^2^)

**Table:**

	*U*^11^	*U*^22^	*U*^33^	*U*^12^	*U*^13^	*U*^23^
O1	0.0306 (7)	0.0246 (8)	0.0275 (8)	−0.0040 (6)	0.0093 (6)	−0.0023 (6)
O2	0.0342 (8)	0.0240 (8)	0.0278 (7)	−0.0030 (7)	0.0073 (6)	−0.0033 (7)
O3	0.0278 (7)	0.0373 (9)	0.0275 (8)	0.0075 (7)	0.0080 (6)	0.0036 (7)
O4	0.0257 (7)	0.0295 (8)	0.0284 (8)	0.0054 (6)	0.0117 (6)	0.0017 (7)
C1	0.0180 (8)	0.0254 (10)	0.0247 (9)	−0.0014 (7)	0.0067 (7)	−0.0023 (8)
N2	0.0234 (8)	0.0222 (9)	0.0235 (8)	−0.0025 (7)	0.0089 (6)	−0.0006 (7)
C3	0.0259 (10)	0.0221 (11)	0.0247 (9)	−0.0005 (8)	0.0090 (7)	−0.0014 (8)
C3A	0.0207 (9)	0.0244 (10)	0.0240 (9)	0.0004 (8)	0.0072 (7)	−0.0010 (8)
C4	0.0207 (8)	0.0225 (10)	0.0250 (9)	0.0007 (8)	0.0079 (7)	−0.0012 (8)
C4A	0.0196 (8)	0.0231 (10)	0.0248 (10)	0.0009 (8)	0.0060 (7)	0.0020 (8)
O5	0.0270 (7)	0.0233 (8)	0.0259 (7)	0.0006 (6)	0.0093 (5)	0.0000 (6)
C5A	0.0213 (9)	0.0273 (11)	0.0228 (10)	0.0018 (8)	0.0064 (7)	−0.0008 (8)
C6	0.0269 (10)	0.0260 (11)	0.0301 (11)	0.0006 (8)	0.0073 (8)	0.0014 (9)
C7	0.0309 (11)	0.0319 (13)	0.0253 (10)	0.0015 (9)	0.0086 (8)	0.0053 (9)
C8	0.0316 (10)	0.0338 (13)	0.0240 (10)	0.0025 (9)	0.0106 (8)	0.0008 (9)
C9	0.0296 (10)	0.0263 (11)	0.0250 (10)	0.0023 (9)	0.0090 (8)	−0.0006 (9)
C9A	0.0195 (8)	0.0259 (11)	0.0234 (10)	0.0020 (8)	0.0059 (7)	0.0011 (8)
C9B	0.0199 (8)	0.0234 (10)	0.0219 (9)	0.0018 (7)	0.0052 (7)	−0.0005 (8)
C10	0.0214 (9)	0.0222 (10)	0.0227 (9)	0.0004 (7)	0.0080 (7)	−0.0001 (8)
C10A	0.0189 (8)	0.0238 (10)	0.0244 (9)	0.0000 (8)	0.0061 (7)	−0.0014 (8)
C11	0.0192 (8)	0.0279 (11)	0.0212 (9)	−0.0020 (8)	0.0060 (7)	−0.0001 (8)
C12	0.0233 (9)	0.0242 (11)	0.0271 (10)	0.0010 (8)	0.0076 (7)	−0.0008 (9)
C13	0.0219 (9)	0.0285 (11)	0.0245 (10)	−0.0013 (8)	0.0070 (7)	−0.0004 (9)
C14	0.0237 (9)	0.0286 (12)	0.0246 (10)	−0.0031 (8)	0.0065 (7)	0.0003 (8)
C15	0.0229 (9)	0.0243 (11)	0.0275 (10)	−0.0026 (8)	0.0043 (7)	−0.0004 (9)
C16	0.0207 (9)	0.0248 (11)	0.0247 (9)	−0.0013 (7)	0.0073 (7)	−0.0021 (8)
C17	0.0372 (12)	0.0322 (13)	0.0253 (10)	0.0051 (10)	0.0119 (8)	0.0004 (9)
C18	0.0354 (11)	0.0266 (12)	0.0307 (11)	0.0019 (9)	0.0079 (8)	0.0002 (9)
C19	0.0220 (9)	0.0227 (10)	0.0267 (10)	−0.0020 (8)	0.0073 (7)	−0.0020 (8)
C20	0.0267 (10)	0.0274 (11)	0.0250 (10)	−0.0017 (8)	0.0059 (7)	−0.0023 (9)
O6	0.0307 (8)	0.0297 (9)	0.0369 (8)	0.0032 (7)	0.0135 (6)	0.0011 (7)
N1	0.0236 (8)	0.0310 (11)	0.0305 (9)	0.0014 (8)	0.0015 (7)	−0.0003 (8)
C21	0.0250 (10)	0.0289 (12)	0.0287 (10)	0.0006 (9)	0.0059 (8)	−0.0017 (9)
C22	0.0318 (11)	0.0363 (14)	0.0328 (11)	0.0102 (10)	0.0023 (8)	−0.0057 (10)
C23	0.0305 (11)	0.0341 (13)	0.0463 (14)	−0.0025 (10)	−0.0030 (10)	0.0077 (11)

### (3aRS,4RS,10SR,10aSR)-2-(3,5-Dimethylphenyl)-4-hydroxy-10-methyl-1-oxo-2,3,3a,4,10,10a-hexahydro-1H-[1]benzofuro[2,3-f]isoindole-10-carboxylic acid dimethylformamide monosolvate Geometric parameters (Å, º)

**Table:**

O1—C1	1.221 (3)	C10—C10A	1.543 (3)
O2—C4	1.423 (3)	C10—C20	1.545 (3)
O2—H2O	0.99 (4)	C10A—H10A	1.0000
O3—C19	1.204 (3)	C11—C16	1.393 (3)
O4—C19	1.329 (3)	C11—C12	1.402 (3)
O4—H4O	0.85 (4)	C12—C13	1.397 (3)
C1—N2	1.369 (3)	C12—H12	0.9500
C1—C10A	1.525 (3)	C13—C14	1.388 (4)
N2—C11	1.422 (3)	C13—C17	1.513 (3)
N2—C3	1.475 (3)	C14—C15	1.394 (3)
C3—C3A	1.528 (3)	C14—H14	0.9500
C3—H3A	0.9900	C15—C16	1.404 (3)
C3—H3B	0.9900	C15—C18	1.498 (3)
C3A—C4	1.517 (3)	C16—H16	0.9500
C3A—C10A	1.525 (3)	C17—H17A	0.9800
C3A—H3C	1.0000	C17—H17B	0.9800
C4—C4A	1.502 (3)	C17—H17C	0.9800
C4—H4	1.0000	C18—H18A	0.9800
C4A—C9B	1.356 (3)	C18—H18B	0.9800
C4A—O5	1.373 (3)	C18—H18C	0.9800
O5—C5A	1.382 (3)	C20—H20A	0.9800
C5A—C6	1.385 (3)	C20—H20B	0.9800
C5A—C9A	1.398 (3)	C20—H20C	0.9800
C6—C7	1.391 (3)	O6—C21	1.242 (3)
C6—H6	0.9500	N1—C21	1.324 (3)
C7—C8	1.401 (4)	N1—C23	1.444 (4)
C7—H7	0.9500	N1—C22	1.464 (3)
C8—C9	1.391 (3)	C21—H21	0.9500
C8—H8	0.9500	C22—H22A	0.9800
C9—C9A	1.407 (3)	C22—H22B	0.9800
C9—H9	0.9500	C22—H22C	0.9800
C9A—C9B	1.453 (3)	C23—H23A	0.9800
C9B—C10	1.529 (3)	C23—H23B	0.9800
C10—C19	1.533 (3)	C23—H23C	0.9800
			
C4—O2—H2O	108 (2)	C3A—C10A—H10A	106.6
C19—O4—H4O	104 (3)	C1—C10A—H10A	106.6
O1—C1—N2	126.1 (2)	C10—C10A—H10A	106.6
O1—C1—C10A	127.2 (2)	C16—C11—C12	119.8 (2)
N2—C1—C10A	106.56 (19)	C16—C11—N2	121.94 (19)
C1—N2—C11	126.5 (2)	C12—C11—N2	118.1 (2)
C1—N2—C3	113.19 (18)	C13—C12—C11	120.5 (2)
C11—N2—C3	120.22 (18)	C13—C12—H12	119.7
N2—C3—C3A	102.03 (17)	C11—C12—H12	119.7
N2—C3—H3A	111.4	C14—C13—C12	118.9 (2)
C3A—C3—H3A	111.4	C14—C13—C17	120.8 (2)
N2—C3—H3B	111.4	C12—C13—C17	120.2 (2)
C3A—C3—H3B	111.4	C13—C14—C15	121.5 (2)
H3A—C3—H3B	109.2	C13—C14—H14	119.3
C4—C3A—C10A	110.85 (18)	C15—C14—H14	119.3
C4—C3A—C3	117.16 (18)	C14—C15—C16	119.2 (2)
C10A—C3A—C3	102.90 (17)	C14—C15—C18	120.8 (2)
C4—C3A—H3C	108.5	C16—C15—C18	119.9 (2)
C10A—C3A—H3C	108.5	C11—C16—C15	119.9 (2)
C3—C3A—H3C	108.5	C11—C16—H16	120.0
O2—C4—C4A	111.45 (17)	C15—C16—H16	120.0
O2—C4—C3A	109.92 (17)	C13—C17—H17A	109.5
C4A—C4—C3A	105.01 (18)	C13—C17—H17B	109.5
O2—C4—H4	110.1	H17A—C17—H17B	109.5
C4A—C4—H4	110.1	C13—C17—H17C	109.5
C3A—C4—H4	110.1	H17A—C17—H17C	109.5
C9B—C4A—O5	113.02 (18)	H17B—C17—H17C	109.5
C9B—C4A—C4	129.1 (2)	C15—C18—H18A	109.5
O5—C4A—C4	117.87 (19)	C15—C18—H18B	109.5
C4A—O5—C5A	105.24 (17)	H18A—C18—H18B	109.5
O5—C5A—C6	124.4 (2)	C15—C18—H18C	109.5
O5—C5A—C9A	110.75 (19)	H18A—C18—H18C	109.5
C6—C5A—C9A	124.8 (2)	H18B—C18—H18C	109.5
C5A—C6—C7	115.8 (2)	O3—C19—O4	123.6 (2)
C5A—C6—H6	122.1	O3—C19—C10	124.22 (19)
C7—C6—H6	122.1	O4—C19—C10	112.22 (18)
C6—C7—C8	121.4 (2)	C10—C20—H20A	109.5
C6—C7—H7	119.3	C10—C20—H20B	109.5
C8—C7—H7	119.3	H20A—C20—H20B	109.5
C9—C8—C7	121.6 (2)	C10—C20—H20C	109.5
C9—C8—H8	119.2	H20A—C20—H20C	109.5
C7—C8—H8	119.2	H20B—C20—H20C	109.5
C8—C9—C9A	118.2 (2)	C21—N1—C23	122.0 (2)
C8—C9—H9	120.9	C21—N1—C22	119.1 (2)
C9A—C9—H9	120.9	C23—N1—C22	118.7 (2)
C5A—C9A—C9	118.2 (2)	O6—C21—N1	123.6 (2)
C5A—C9A—C9B	105.37 (19)	O6—C21—H21	118.2
C9—C9A—C9B	136.4 (2)	N1—C21—H21	118.2
C4A—C9B—C9A	105.6 (2)	N1—C22—H22A	109.5
C4A—C9B—C10	122.89 (18)	N1—C22—H22B	109.5
C9A—C9B—C10	131.4 (2)	H22A—C22—H22B	109.5
C9B—C10—C19	111.18 (16)	N1—C22—H22C	109.5
C9B—C10—C10A	105.37 (18)	H22A—C22—H22C	109.5
C19—C10—C10A	109.91 (17)	H22B—C22—H22C	109.5
C9B—C10—C20	111.64 (18)	N1—C23—H23A	109.5
C19—C10—C20	107.81 (18)	N1—C23—H23B	109.5
C10A—C10—C20	110.95 (17)	H23A—C23—H23B	109.5
C3A—C10A—C1	103.62 (17)	N1—C23—H23C	109.5
C3A—C10A—C10	113.22 (17)	H23A—C23—H23C	109.5
C1—C10A—C10	119.38 (19)	H23B—C23—H23C	109.5
			
O1—C1—N2—C11	−1.1 (3)	C9A—C9B—C10—C10A	166.1 (2)
C10A—C1—N2—C11	−177.07 (19)	C4A—C9B—C10—C20	−131.6 (2)
O1—C1—N2—C3	175.4 (2)	C9A—C9B—C10—C20	45.5 (3)
C10A—C1—N2—C3	−0.6 (2)	C4—C3A—C10A—C1	158.47 (16)
C1—N2—C3—C3A	21.1 (2)	C3—C3A—C10A—C1	32.4 (2)
C11—N2—C3—C3A	−162.14 (17)	C4—C3A—C10A—C10	−70.7 (2)
N2—C3—C3A—C4	−154.02 (18)	C3—C3A—C10A—C10	163.22 (18)
N2—C3—C3A—C10A	−32.2 (2)	O1—C1—C10A—C3A	163.7 (2)
C10A—C3A—C4—O2	167.45 (16)	N2—C1—C10A—C3A	−20.4 (2)
C3—C3A—C4—O2	−74.9 (2)	O1—C1—C10A—C10	36.7 (3)
C10A—C3A—C4—C4A	47.5 (2)	N2—C1—C10A—C10	−147.41 (18)
C3—C3A—C4—C4A	165.10 (17)	C9B—C10—C10A—C3A	46.8 (2)
O2—C4—C4A—C9B	−132.0 (2)	C19—C10—C10A—C3A	−73.1 (2)
C3A—C4—C4A—C9B	−13.1 (3)	C20—C10—C10A—C3A	167.77 (19)
O2—C4—C4A—O5	45.1 (2)	C9B—C10—C10A—C1	169.18 (17)
C3A—C4—C4A—O5	164.06 (17)	C19—C10—C10A—C1	49.3 (2)
C9B—C4A—O5—C5A	0.6 (2)	C20—C10—C10A—C1	−69.8 (2)
C4—C4A—O5—C5A	−176.98 (17)	C1—N2—C11—C16	−32.0 (3)
C4A—O5—C5A—C6	177.6 (2)	C3—N2—C11—C16	151.7 (2)
C4A—O5—C5A—C9A	−0.5 (2)	C1—N2—C11—C12	151.8 (2)
O5—C5A—C6—C7	−178.00 (19)	C3—N2—C11—C12	−24.5 (3)
C9A—C5A—C6—C7	−0.2 (3)	C16—C11—C12—C13	−2.5 (3)
C5A—C6—C7—C8	0.3 (3)	N2—C11—C12—C13	173.85 (19)
C6—C7—C8—C9	−0.3 (4)	C11—C12—C13—C14	0.2 (3)
C7—C8—C9—C9A	0.0 (4)	C11—C12—C13—C17	−179.54 (19)
O5—C5A—C9A—C9	178.00 (17)	C12—C13—C14—C15	1.6 (3)
C6—C5A—C9A—C9	−0.1 (3)	C17—C13—C14—C15	−178.6 (2)
O5—C5A—C9A—C9B	0.2 (2)	C13—C14—C15—C16	−1.2 (3)
C6—C5A—C9A—C9B	−177.9 (2)	C13—C14—C15—C18	178.4 (2)
C8—C9—C9A—C5A	0.2 (3)	C12—C11—C16—C15	2.9 (3)
C8—C9—C9A—C9B	177.2 (2)	N2—C11—C16—C15	−173.26 (19)
O5—C4A—C9B—C9A	−0.5 (2)	C14—C15—C16—C11	−1.1 (3)
C4—C4A—C9B—C9A	176.74 (19)	C18—C15—C16—C11	179.3 (2)
O5—C4A—C9B—C10	177.23 (18)	C9B—C10—C19—O3	−123.8 (2)
C4—C4A—C9B—C10	−5.5 (3)	C10A—C10—C19—O3	−7.5 (3)
C5A—C9A—C9B—C4A	0.2 (2)	C20—C10—C19—O3	113.6 (2)
C9—C9A—C9B—C4A	−177.0 (2)	C9B—C10—C19—O4	56.9 (2)
C5A—C9A—C9B—C10	−177.3 (2)	C10A—C10—C19—O4	173.20 (19)
C9—C9A—C9B—C10	5.5 (4)	C20—C10—C19—O4	−65.7 (2)
C4A—C9B—C10—C19	108.0 (2)	C23—N1—C21—O6	179.5 (2)
C9A—C9B—C10—C19	−74.9 (3)	C22—N1—C21—O6	4.0 (3)
C4A—C9B—C10—C10A	−11.0 (3)		

### (3aRS,4RS,10SR,10aSR)-2-(3,5-Dimethylphenyl)-4-hydroxy-10-methyl-1-oxo-2,3,3a,4,10,10a-hexahydro-1H-[1]benzofuro[2,3-f]isoindole-10-carboxylic acid dimethylformamide monosolvate Hydrogen-bond geometry (Å, º)

*Cg*4 and *Cg*5 are the centroids of the C5*A*/C6–C9/C9*A* and C11–C16 rings, respectively.

**Table:**

*D*—H···*A*	*D*—H	H···*A*	*D*···*A*	*D*—H···*A*
O2—H2*O*···O1^i^	0.99 (5)	2.23 (4)	3.013 (2)	135 (3)
O2—H2*O*···O3^i^	0.99 (5)	2.32 (4)	3.152 (3)	141 (3)
O4—H4*O*···O6	0.84 (4)	1.74 (4)	2.560 (3)	165 (4)
C3—H3*A*···O1^ii^	0.99	2.54	3.452 (3)	152
C8—H8···O6^iii^	0.95	2.44	3.344 (3)	159
C16—H16···O1	0.95	2.44	2.970 (3)	115
C20—H20*B*···O1	0.98	2.59	3.229 (3)	123
C10*A*—H10*A*···*Cg*5^ii^	1.00	2.88	3.865 (2)	169
C22—H22*A*···*Cg*5^iv^	0.98	2.96	3.401 (3)	109
C22—H22*C*···*Cg*4^v^	0.98	2.68	3.594 (3)	155

Symmetry codes: (i) *x*, *y*−1, *z*; (ii) −*x*+2, *y*−1/2, −*z*+1; (iii) −*x*+1, *y*−1/2, −*z*+2; (iv) −*x*+1, *y*+1/2, −*z*+1; (v) *x*, *y*+1, *z*.
